# *ZmACY-1* Antagonistically Regulates Growth and Stress Responses in *Nicotiana benthamiana*

**DOI:** 10.3389/fpls.2021.593001

**Published:** 2021-07-23

**Authors:** Dongbin Chen, Junhua Li, Fuchao Jiao, Qianqian Wang, Jun Li, Yuhe Pei, Meiai Zhao, Xiyun Song, Xinmei Guo

**Affiliations:** ^1^College of Life Sciences, Qingdao Agricultural University, Qingdao, China; ^2^Key Laboratory of Qingdao Major Crop Germplasm Resource Innovation and Application, Qingdao, China; ^3^College of Agronomy, Qingdao Agricultural University, Qingdao, China

**Keywords:** aminoacylase-1, salt stress, drought stress, plant growth, trade-off, *Nicotiana benthamiana*

## Abstract

Aminoacylase-1 is a zinc-binding enzyme that is important in urea cycling, ammonia scavenging, and oxidative stress responses in animals. Aminoacylase-1 (*ACY-1*) has been reported to play a role in resistance to pathogen infection in the model plant *Nicotiana benthamiana*. However, little is known about its function in plant growth and abiotic stress responses. In this study, we cloned and analyzed expression patterns of *ZmACY-1* in *Zea mays* under different conditions. We also functionally characterized *ZmACY-1* in *N. benthamiana*. We found that *ZmACY-1* is expressed specifically in mature shoots compared with other tissues. *ZmACY-1* is repressed by salt, drought, jasmonic acid, and salicylic acid, but is induced by abscisic acid and ethylene, indicating a potential role in stress responses and plant growth. The overexpression of *ZmACY-1* in *N. benthamiana* promoted growth rate by promoting growth-related genes, such as *NbEXPA1* and *NbEIN2*. At the same time, the overexpression of *ZmACY-1* in *N. benthamiana* reduced tolerance to drought and salt stress. With drought and salt stress, the activity of protective enzymes, such as peroxidase (POD), superoxide dismutase (SOD), and catalase (CAT) from micrococcus lysodeikticus was lower; while the content of malondialdehyde (MDA) and relative electrolytic leakage was higher in *ZmACY-1* overexpression lines than that in wild-type lines. The results indicate that *ZmACY-1* plays an important role in the balance of plant growth and defense and can be used to assist plant breeding under abiotic stress conditions.

## Introduction

Water limitation, normally caused by drought and salt stress, is one of the most important factors affecting plant growth (Koevoets et al., 2016). Drought induces a series of changes at the morphological, physiological, and biochemical levels. Drought leads to stomatal closure; mechanical damage to protoplasts, cell walls, and biofilm systems; decline in photosynthetic efficiency and cell dehydration, which will eventually lead to metabolic disorder (Gupta et al., 2020). Salt stress leads to extracellular osmotic stress, ionic toxicity, production of reactive oxygen species (ROS), and accumulation of superoxide anions, thus disrupting ion balance and stability of biological macromolecules (Claeys and Inzé, 2013). Climate change threatens to exacerbate the problem of water limitation on plant growth and food production (Van Zelm et al., 2020).

To understand the physiological and molecular mechanisms of plant growth under stressed conditions, several signaling pathways have been elucidated (Van Wallendael et al., 2019). For example, DELLAS (Lantzouni et al., 2020) and AP2/ERF-type transcriptional factors (Xie et al., 2019) have been demonstrated as crucial regulators in plant growth and stress tolerance. In addition, amino acid metabolism, such as glutathione, arginine, and proline, have been reported to play important roles in stress responses. Glutathione is composed of glutamic acid, cysteine, and glycine; and glutathione reductase (GR) accumulates in salt-tolerant pea lines with long-term salt stress (Hernandez et al., 2000). Glutathione S-transferase regulates stress-activated signals by inhibiting the activity of apoptotic signal kinase 1 (Cho et al., 2001). High expression of glutathione S-transferase promotes the growth of tobacco seedlings under stress conditions (Roxas et al., 1997). Knocking out the arginine metabolism-related gene *AtARG2* enhanced environmental tolerance (Shi et al., 2013). Proline protects macromolecules and cell membranes during dehydration and also acts as an osmotic agent and free radical scavenger. The accumulation of proline is involved in the scavenging of ROS (Schwörer et al., 2020).

Aminoacylase-1 is a homodimer zinc-binding enzyme in cell solutes, which catalyzes the hydrolysis of acylated L-amino acids to L-amino acids and acyl groups. It plays a role in the catabolism and recovery of acylated amino acids. N-acetyl-L-glutamic acid is an allosteric activator of carbamyl phosphate synthase, and carbamyl phosphate synthase is a key enzyme that converts NH_4_^+^ molecules in the urea cycle. When amino acid catabolism increases, N-acetylglutamate synthase is up-regulated to produce more N-acetyl-L-glutamate, which up-regulates carbamyl phosphate synthase and allows it to deal with excess NH_4_^+^. In addition, *ACY-1* may play a role in regulating the cellular response to oxidative stress in animals by interacting with sphingosine kinase-1 (Maceyka et al., 2004).

Currently, aminoacylase-1 (DS2) has been found to play a role in plant defense systems and in the salicylic acid-dependent pathway. Silencing *DS2* enhanced the resistance of *Nicotiana benthamiana* to *Phytophthora infestans* (Nakano et al., 2014). However, the functional importance of aminoacylase-1 in plant growth and abiotic stress responses is still unknown. In this study, we cloned *ZmACY-1* and transformed it into the model plant *N. benthamiana*. We found an important role of *ZmACY-1* in plant growth and stress resistance. This study highlights amino acid metabolism as a new way for improving plant performance in stressed conditions.

## Results

### Characterization of Aminoacylase-1 Homologs

First, in order to clone *ZmACY-1*, we analyzed its sequence *in silico* using the Bioxm software. The full-length open reading frame of *ZmACY-1* is 1,317 base pairs, and it encodes 439 amino acids. The molecular formula of *ZmACY-1* is C_2173_H_3367_N_599_O_625_S_14_, the relative molecular weight is 48.33 kDa, the theoretical isoelectric point is 6.02, the total number of positive charge residues (Arg + Lys) is 42, the total number of negative charge residues (Asp + Glu) is 50, and the coefficient of instability is 46.03. *ZmACY-1* belongs to the zinc peptidase superfamily and M20 aminoacylase-1 subfamily, and it contains an M20-acylase domain and five zinc binding sites.

**FIGURE 1:**
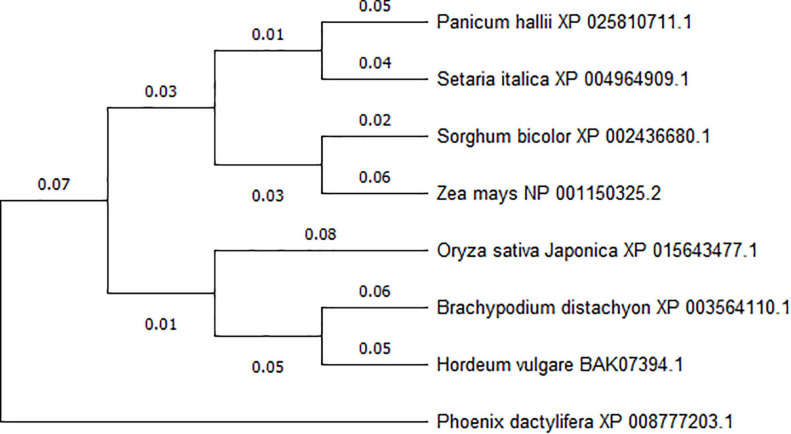
Phylogenetic analysis of ACY-1 based on amino acid sequences. A phylogenetic tree was generated from the deduced amino acid sequences of plant ACY-1 genes. Phylogenetic relationships were calculated using the maximum likelihood method based on DNAMAN and MEGA 5.1 software.

To investigate the conservation of *ACY-1* between different plant species, we used NCBI Blast to search for homologous amino acid sequences and used the MEGA 5.1 software for phylogenetic analysis. We found that the *ZmACY-1* amino acid sequence (NP_001150325.2) and *SbACY-1* amino acid sequence (XP_002436680.1) in *Sorghum bicolor* showed up to 92.26% similarity. In addition, *ZmACY-1* is closely related to *SiACY-1* and *PhACY-1* (Figure 1), indicating that *ACY-1* is highly conserved in plants.

### Expression Patterns of *ZmACY-1*

**FIGURE 2:**
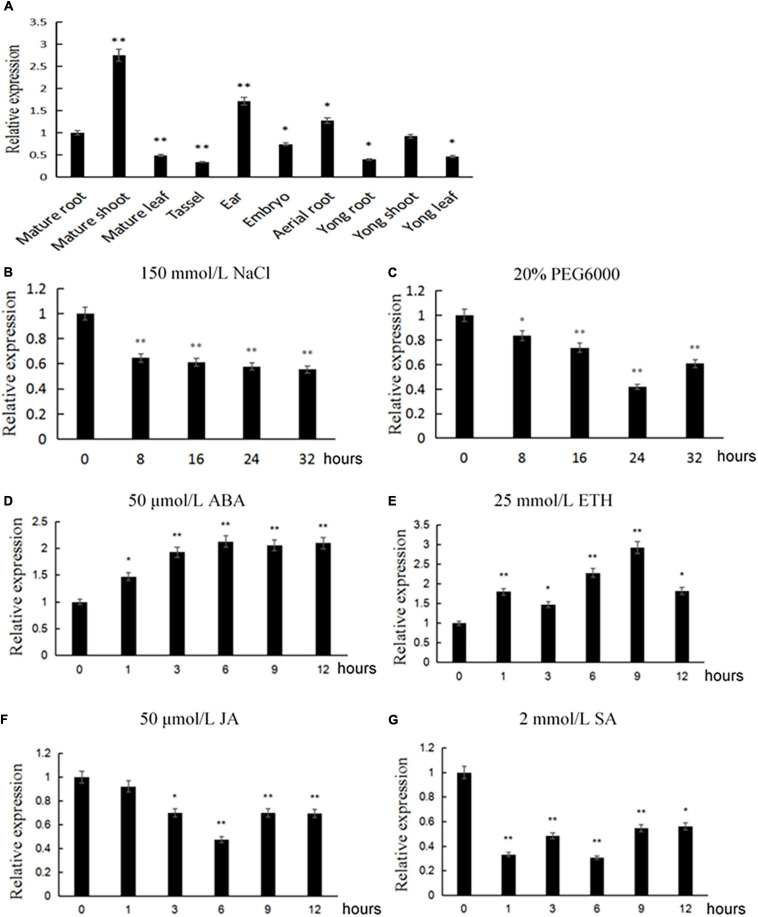
Expression pattern of *ZmACY-1.* The relative expression of *ZmACY-1* under different conditions was measured. **(A)** Different tissues; **(B)** 150 mM NaCl; **(C)** 20% PEG 6000; **(D)** 50 μM abscisic acid; **(G)** 2 mM salicylic acid; **(F)** 50 μM jasmonic acid; **(E)** 25 mM ethylene. Three biological replicates were used. *P < 0.05, **P < 0.01.

In order to detect the expression characteristics of *ZmACY-1* in different stages and tissues of maize, we performed fluorescence quantitative PCR analysis on young roots, young stems, and young leaves of the trifoliate stage; mature roots, mature stems, mature leaves, aerial roots, tassel, ears of heading stage; and immature embryos 14 days after pollination (DAP). We found that the expression of *ZmACY-1* was both tissue- and stage-specific (Figure 2A). *ZmACY-1* is expressed highest in mature shoots where the expression of *ZmACY-1* is about two times higher than that in young roots and young leaves, 2.75 times higher than that in mature roots, and 5.5 times higher than that in mature leaves. The fact that *ZmACY-1* is most highly expressed in shoots indicates that *ZmACY-1* is associated with plant growth rate. *ZmACY-1* is also expressed in ears, indicating that it may also be involved in plant development.

Since *ACY-1* has an important role in response to *Phytophthora infestans*, we tested the expression of *ZmACY-1* with different stress treatments in order to explore whether *ZmACY-1* is affected by abiotic stress or hormone regulation. We treated trifoliate-stage maize seedlings with 150 mM NaCl, 20% PEG6000, and plant defense-related hormones (50 μM abscisic acid, 2 mM salicylic acid, 50 μM jasmonic acid, and 25 mM ethylene). We found that 150 mM NaCl (Figure 2B), 20% PEG6000 (Figure 2C), jasmonic acid (Figure 2F), and salicylic acid (Figure 2G) decreased the expression of *ZmACY-1*, while ABA (Figure 2D) and ethylene (Figure 2E) increased the expression of *ZmACY-1*. This suggested that *ZmACY-1* may be involved in defense responses to drought stress, salt stress, and various plant hormones.

### Functional Characterization of *ZmACY-1* in Plant Growth

**FIGURE 3:**
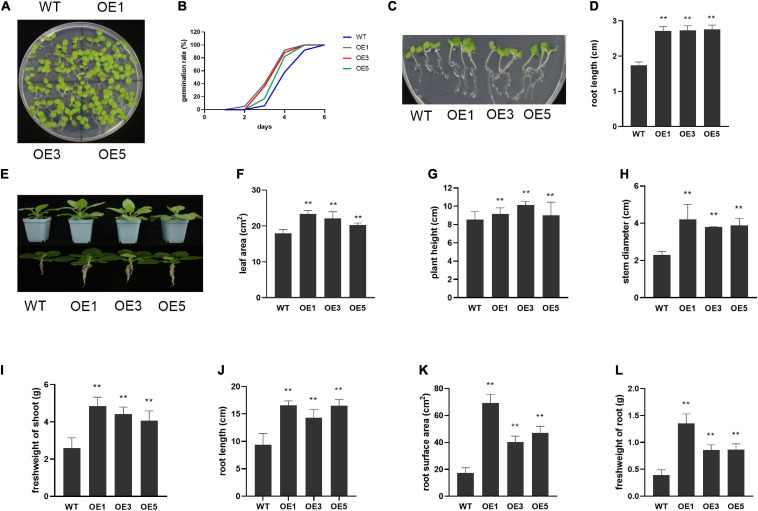
Function of *ZmACY-1* in plant growth. **(A)** Ten-day-old *N. benthamiana* in MS-medium-supplemented plate; **(B)** Germination rate in MS-medium-supplemented plate; **(C,D)** Root length; **(E–L)** One-month-old *N. benthamiana* in pods. **(F)** Leaf area; **(G)** Plant height; **(H)** Stem diameter; **(I)** Shoot fresh weight; **(J)** Root length; **(K)** Root surface area; **(L)** Fresh weight of roots. Three biological replicates were used. *P < 0.05, **P < 0.01.

To further reveal the function of *ZmACY-1* in plant growth, we constructed plasmids and generated transgenic lines in *N. benthamiana.* We obtained three independent transgenic lines, and the expression of ZmACY-1 in all the three transgenic lines was significantly higher than that in the wild type (Supplementary Figure 1). Then, these transgenic lines were phenotyped for yield-associated traits, such as germination, plant height, root length, stem diameter, leaf area, root area, shoot fresh weight, and fresh weight of roots and pods (Figure 3). We found that seed germination rate is higher in *ZmACY-1* overexpression lines than in wild-type lines. The germination rate in transgenic lines reached 80–90% after 4 days, while wild-type controls only reached 60% germination (Figures 3A,B). In addition, the root length, leaf area, plant height, stem diameter, shoot fresh weight, and root fresh weight of the transgenic lines all overmatched those of the wild type (Figures 3E–L), which indicates that overexpression of *ZmACY-1* promotes growth of *N. benthamiana*.

**FIGURE 4:**
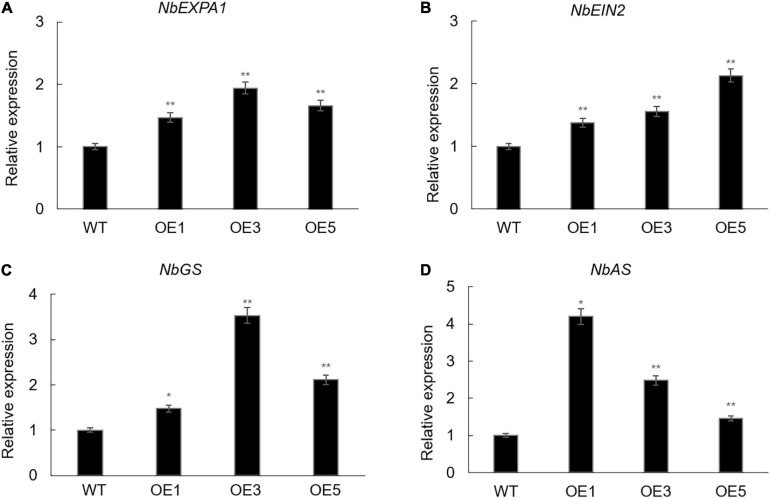
Downstream gene targets of *ZmACY-1.* **(A)** Relative expression of *NbEXPA1*; **(B)** Relative expression of *NbEIN2*; **(C)** Relative expression of *NbGS*; **(D)** Relative expression of *NbAS*. Three biological replicates were used. *P < 0.05, **P < 0.01.

The *EXPA* gene family plays an important role in promoting leaf growth (Goh et al., 2012). *Ein2* has been proved to be a positive regulator in the ethylene signaling pathway, which participates in many aspects of the plant life cycle (Li et al., 2015). Glutamine synthetase (GS) and asparagine synthetase (AS) have important roles in nitrogen metabolism (Németh et al., 2018; Rashmi et al., 2019). Thus, in order to study whether the overexpression of *ZmACY-1* in *N. benthamiana* promoted the expression of plant growth-related and nitrogen assimilation-related genes, we detected the expression levels of *NbEXPA1*, *NbEIN2*, *NbGS*, and *NbAS* (Figure 4). We found that the expression levels of *NbEXPA1*, *NbEIN2*, *NbGS*, and *NbAS* in transgenic *N. benthamiana* were significantly higher than those in the wild type, indicating that *ZmACY-1* functions by increasing the expression of growth-related and nitrogen assimilation-related genes.

**FIGURE 5:**
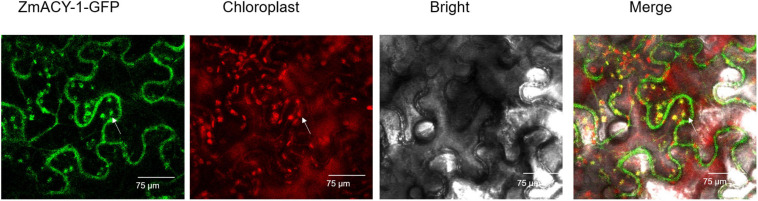
ZmACY-1 accumulates in the chloroplasts. Transient expression of ZmACY-1:GFP in *N. benthamiana* leaves. The full-length cDNA encoding *ZmACY-1* was fused into the pCambia1300-GFP vector. The white arrow illustrates the overlap between ZmACY-1-GFP and the chloroplast signal.

It is predicted by pLoc-mPlant that the ZmACY-1 protein is mainly located in the chloroplast. Compared with the green fluorescence of the empty vector, the green fluorescence of the pCambia1300-ZmACY-1 fusion protein can be seen in both the cell membrane and the chloroplast, indicating that the ZmACY-1 protein plays a role in the cell membrane and chloroplast (Figure 5).

### Functional Characterization of *ZmACY-1* in Abiotic Stress

**FIGURE 6:**
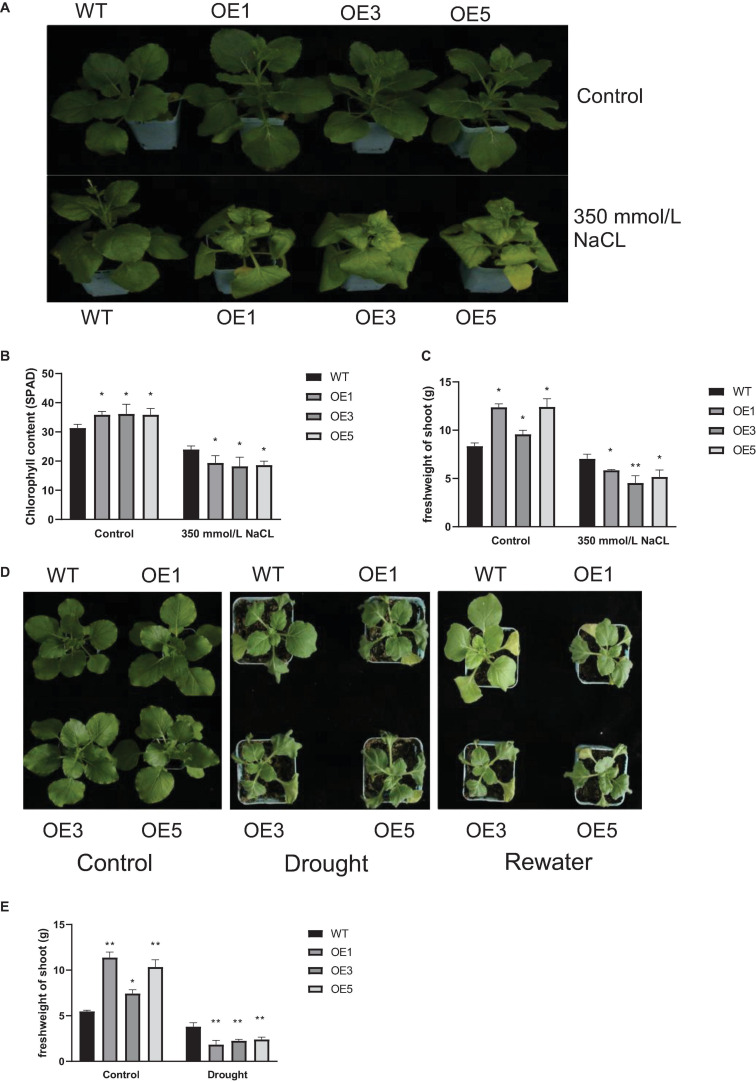
Function of *ZmACY-1* in stress responses. **(A)** Phenotype of *N. benthamiana* under 350 mM NaCl stress treatment; **(B)** Chlorophyll content; **(C)** Shoot fresh weight; **(D)** Phenotype of *N. benthamiana* with drought treatment; **(E)** Shoot fresh weight. Three biological replicates were used. *P < 0.05, **P < 0.01.

To further determine the function of *ZmACY-1*, we tested the performance of transgenic *N. benthamiana* under stress conditions. First, we performed a salt stress treatment by growing 1-month-old *N. benthamiana* in a 350 mM NaCl solution. We found that after 10 days of salt treatment, the transgenic plants exhibited a higher degree of chlorosis than the wild-type plants (Figure 6A). Under normal conditions, the chlorophyll content (Figure 6B) and shoot fresh weight (Figure 6C) in transgenic lines were significantly higher than those of the wild type; while after 10 days of salt stress, the chlorophyll content and shoot fresh weight of the transgenic lines were significantly lower than those of the wild type. This indicated that the overexpression of *ZmACY-1* in *N. benthamiana* reduced salt stress tolerance.

Then, we performed drought treatment by withholding water on 1-month-old *N. benthamiana*. We found that after 7 days of drought treatment, the wilting degree of transgenic lines was more serious than that of the wild type, and that the recovery degree of the transgenic lines was significantly lower than that of the wild type (Figure 6D). Drought stress significantly reduced the shoot fresh weight of the wild-type and transgenic lines (Figure 6E). The shoot fresh weight of transgenic lines was significantly higher than that of the wild type under normal conditions. However, with drought stress, the shoot fresh weight was significantly lower than that of the wild type. These results showed that the overexpression of *ZmACY-1* in *N. benthamiana* reduced drought stress resistance.

**FIGURE 7:**
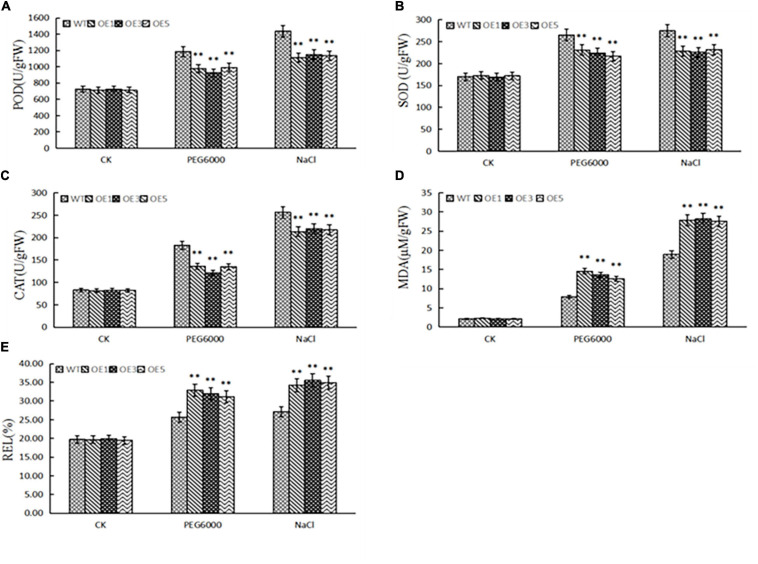
Physiological indices related to salt and drought stress. **(A)** POD activity; **(B)** SOD activity; **(C)** CAT activity; **(D)** MDA activity; **(E)** Electrolytic leakage. Three biological replicates were used. *P < 0.05, **P < 0.01.

To determine the physiological mechanisms, we tested the contents of peroxidase (POD), superoxide dismutase (SOD), catalase (CAT) from micrococcus lysodeikticus, and malondialdehyde (MDA), and relative electrolytic leakage after 2 days of treatment with water, 20% PEG6000, and 350 mM NaCl. We found that drought and salt stress increased POD, SOD, and CAT activities, MDA content, and electrolytic leakage (Figure 7). However, with drought and salt stress, the activities of POD, SOD, and CAT in the transgenic lines were lower, and MDA content and electrolytic leakage was higher than those of the wild type. This further revealed that *ZmACY-1* is involved in the process of stress responses.

## Discussion

Aminoacylase-1 is a dimer zinc-binding enzyme that can catalyze the hydrolysis of acylated L-amino acids into L-amino acids and acyl groups, and is considered to play a role in the catabolism and recovery of acylated amino acids. In this study, we provided experimental evidence for the function of *ZmACY-1* in plants. The finding that *ZmACY-1* promotes plant growth while reducing stress tolerance may be a new signaling pathway for plant growth-defense trade-off.

The tissue and spatiotemporal specific expression of *ZmACY-1*, and regulation by stress and hormones, indicated that *ZmACY-1* is involved in growth and development in response to environmental changes (Berens et al., 2019). In the trifoliate-stage maize seedlings, *ZmACY-1* is induced by ABA and ethylene while repressed by jasmonic acid and salicylic acid after 3 h; while *ZmACY-1* is repressed by both drought and salt stress after 8 h. This indicates that *ZmACY-1* responds to plant hormones more quickly than salt and drought stress. The transcription levels of hormone genes *NbEIN2*, *NbGS*, and *NbAS* were higher in the *ZmACY-1* transgenic lines than those in the wild type, which is consistent with the function of *EIN2*, *GS*, and *AS* (Li et al., 2015; Németh et al., 2018; Rashmi et al., 2019). *ZmACY-1* might promote plant growth rate and enhance nitrogen assimilation ability by positively regulating the expression of plant growth-related genes. By further elucidating how exactly *ZmACY-1* controls these genes, we can modify plant growth precisely.

Some genes have been identified in plant growth-defense trade-off, such as DELLA, ERF-type TF, and proline. The stability of DELLA promotes stress tolerance but inhibits growth (Verma et al., 2016). Similarly, *ERF6* promotes stress tolerance but inhibits growth through the GA pathway (Dubois et al., 2013). In contrast with *DELLA* and *ERF6*, *ZmACY-1* promotes plant growth under normal conditions. *NEK6* is induced by salt stress stimulating growth (Zhang et al., 2011). Different with *NEK6*, *ZmACY-1* is repressed by salt stresses. Therefore, it may be a new pathway in the growth defense trade-off.

Understanding the signaling pathways in growth-defense trade-off can avoid unnecessary yield loss (Todaka et al., 2015). Aminoacylases are involved in arginine metabolism and methionine (SAM) cycle, which are closely related to stress responses. The data are consistent with the previous study that abiotic stress affects nitrogen assimilation and amino acid metabolism in plants (Charlier and Bervoets, 2019). Under normal conditions, by overexpressing *ZmACY-1*, we can improve plant yield, while under stressed conditions, we have to carefully control the expression of *ZmACY-1* in order to maintain stress tolerance. However, the physiological and molecular mechanisms of *ZmACY-1* in maize growth and abiotic stress responses need to be further revealed (Wang et al., 2018). By constructing plasmids and generating maize transgenic lines, the effects of *ZmACY-1* on plant growth and the mechanism of abiotic stress responses can be verified.

## Experimental Procedures

### Plant Materials and Growth Conditions

Maize inbred line Zheng 58 and *N. benthamiana* were provided by the Crop Breeding Laboratory of Qingdao Agricultural University. Zheng 58 was planted in the field at the Jiaozhou Experimental Station of Qingdao Agricultural University (36° N, 120° E) under normal management. Roots, stems, and leaves of three-leaf stage seedlings; and roots, stems, leaves, tassels, female ears, and aerial roots of mature plants at the heading stage were collected 7 days after pollination for quantitative RT-PCR. *N. benthamiana* was planted in a growth chamber at 23–26°C. All stress and plant hormones treatments were performed at the three-leaf stage. For salt and drought stresses, 150 mM NaCl and 20% PEG6000 were used. Samples were taken after 8, 16, 24, and 32 h of treatment. For the plant hormone treatment, 50 μM abscisic acid, 2 mM salicylic acid, 50 μM jasmonic acid, or 25 mM ethephon was used. Samples were taken after 1, 3, 6, 9, and 12 h of treatment. All the above experiments were repeated three times, with two plants as biological replicates each time.

### Phylogenetic Analysis of *ACY-1* Genes

All sequences were downloaded from the NCBI database. The Bioxm software was used to compare the sequences using default parameters. The MEGA 5.1 software was used to analyze the sequences with phylogenetic tree analysis default parameters.

### Generation of Overexpression Lines in *N. benthamiana*

*ZmACY-1*-*Xba*I-F and *ZmACY-1*-*Bam*HI-R were used as primers for high-fidelity PCR amplification. The pCambia1300 expression vector and PMD19-*ZmACY-1* PCR product were double digested with *Xba*I and *Bam*HI at 37°C for 3 h. Then, the DNA Ligation (Takara, Dalian, China) was used for ligation. The recombinant plasmid was transformed into *Escherichia coli* DH5α and sent to Qingdao QingkeZixi Biotechnology Co., Ltd., (Qingdao, China), for sanger sequencing. The plasmid was transformed into *N. benthamiana* using LBA4404 competent cells. Transgenic *N. benthamiana* lines were incubated and propagated under light at 25°C.

### RNA Isolation, cDNA Generation, and Quantitative RT-PCR

RNA isolation was performed following the Plant RNA Extraction Kit (Takara, Dalian, China) protocol. RNA quality was tested by agarose gel analysis. cDNA was synthesized by master mix kit (Takara, Dalian, China). Quantitative RT-PCR was performed using a TB Green Premix Ex Taq II (Tli RNaseH Plus) kit (Takara, Dalian, China). The endogenous gene *NbEF1a* was used as a reference.

### Phenotyping

Transgenic and wild-type *N. benthamiana* seeds were sown in Petri dishes containing MS medium. Each plate contained 100 seeds. Seed germination rate was measured from the third day. To compare the growth rate of transgenic lines and wild type lines, seeds of each line were aseptically sown on the same plate. Twenty-five seeds were used for each line. Transgenic and wild-type *N. benthamiana* seedlings were cultured in an MS medium for 15 days and then transplanted into soil. After another 15 days, plant height, leaf area, stem thickness, root length, number of leaves, root area, and above-ground weight were measured. Fully developed pods in the middle of the mature stage of tobacco were collected for weighing and fruit length measurement.

#### Protein Subcellular Localization

The pCambia1300-GFP vector was digested with restriction endonucleases *Pst*I (Takara). The CDS sequences of *ZMACY-1* was amplified with gene-specific primer pairs, forward primer: gcttggatcctcgagctgcagATGCCGCCGCCTCTCCGC, reverse primer:acgggtcatgagctcctgcagGCCTTGGAACGAGCTTAGTGC. The PCR products were recovered using the FastPure Gel DNA Extraction Mini Kit (Vazyme Biotech Co., Ltd., Nanjing, China). The *ZmACY-1* gene was connected to the pCambia1300-GFP vector using the ClonExpress II One Step Cloning Kit (Vazyme Biotech Co., Ltd., Nanjing, China) The recombinant plasmids were transferred into *Agrobacterium tumefaciens* strain GV3101. The transformed *A. tumefaciens* was cultured for 24 h at 28°C in L-broth supplemented with 50 μg/ml kanamycin, centrifuged at 5,000 × *g* for 10 min at room temperature and resuspended to a density (OD600) of 1. *A. tumefaciens* cells were infiltrated into the abaxial air spaces of the *N. benthamiana* plants. After 38 h of infiltration, the expression position of the ZMACY-1 proteins was observed with a Leica SP8 laser confocal microscope (Leica Microsystems, Inc., Buffalo Grove, IL, United States) using filter blocks to select for spectral emission at 488 nm.

### Physiology Indexes

Physiology indexes were tested after 350 mM NaCl or 20% PEG6000 treatment.

For MDA, 0.2 g leaves were collected into 3 ml 5% trichloroacetic acid (TCA). After grinding, the mixture was centrifuged at 5,000 rpm for 10 min. The supernatant was transformed in a test tube. An equal volume of 0.5% thiobarbital acid (TBA) was added. After mixing, the samples were incubated in a boiling water bath for 30 min, and absorbance values at 450, 532, and 600 nm were tested. MAD content was calculated using Eq. 1:

(1) MDA⁢content⁢(nM)=6.45×(A532-A600)-0.56×A450

For relative conductivity, 0.2 g leaves were collected in a test tube. Deionized water 20 ml was added until the leaves were immersed. Then, the samples were incubated at room temperature for 4 h. Conductivity R1 was first measured using a conductivity meter. After boiling for 25 min in a water bath, conductivity R2 was measured. Relative conductivity was calculated using Eq. 2:

(2) Relative⁢conductivity=R1/R2×100%

For SOD activity, 0.2 g leaves were collected in a mortar. Pre-chilled 0.1 mol/L Tris–HCL buffer solution 20 ml was added for grinding. Then, the samples were transferred to a 2-ml centrifuge tube and centrifuged at 8,000 rpm for 30 min at 4°C. The supernatant of the crude enzyme solution was stored at −20°C. The SOD activity was measured using the nitrogen blue tetrazole (NBT) method, and the treated crude enzyme liquid sample was measured with a microplate reader at a wavelength of 560 nm. The SOD activity was calculated using Eq. 3:

(3) SODactivity(U/mgprotein)=(A-0A)×VT×(0.5A×0W×V1)

A_0_ is the absorption value of the control tube at 560 nm, A is the absorbance value of the sample tube at 560 nm, VT is the total volume of enzyme extract (ml), V1 is the volume of enzyme solution added during measurement (ml), and W is the fresh weight of sample (g).

The POD activity was measured using the guaiacol colorimetric method. The crude enzyme solution sample was measured for absorbance at 470 nm using a microplate reader, and tested every 1 min for five times in total. The POD activity was calculated using Eq. 4:

(4) POD⁢activity=105×Δ⁢A470/(C×VS×t)

ΔA470 is the change in light absorption within reaction time (t), C is the concentration of protein in enzyme solution (μg/μl), VS is the volume of enzyme solution used for measurement (ml), and t is the reaction time (min).

## Accession Numbers

Sequence data can be found in the NCBI databases under the following accession numbers: *ZmACY-1*: NP_001150325.2, *NbEXPA1*: NM_001325646.1, *NbEIN2*: XM_016579720.1; *NbGS*: XM_016631331.1, and *NbAS*: XM_016658168.1.

## Data Availability Statement

The datasets presented in this study can be found in online repositories. The names of the repository/repositories and accession number(s) can be found in the article/Supplementary Material.

## Author Contributions

DC, XS, and XG designed the study. DC, JL, FJ, and QW performed the experiments. DC, JL, and FJ analyzed the data and wrote the draft manuscript. JL, YP, and MZ discussed and revised the draft manuscript. All authors read and approved the final manuscript.

## Conflict of Interest

The Qingdao Agricultural University has filed a provisional patent application on Aminoacylase-1 in China (202010088971.1) and a provisional patent application on Aminoacylase-1 in Luxembourg (102487) arising from this work.

## Publisher’s Note

All claims expressed in this article are solely those of the authors and do not necessarily represent those of their affiliated organizations, or those of the publisher, the editors and the reviewers. Any product that may be evaluated in this article, or claim that may be made by its manufacturer, is not guaranteed or endorsed by the publisher.

## Supplementary Material

Supplementary Figure 1 Expression patterns of *ZmACY* in overexpressed lines. Wild-type and transgenic *N. benthamiana* were planted in a growth chamber at 23–26°C for 7 days. Three biological replicates were used. ***P* < 0.01.

Supplementary Table 1 Primers used in this study.
