# Supplementary figures and images for: ZmACY-1 Antagonistically Regulates Growth and Stress Responses in Nicotiana benthamiana
Source: Front Plant Sci. 2021 Jul 23;12:593001. doi: 10.3389/fpls.2021.593001 (PMC8343404; doi:10.3389/fpls.2021.593001)

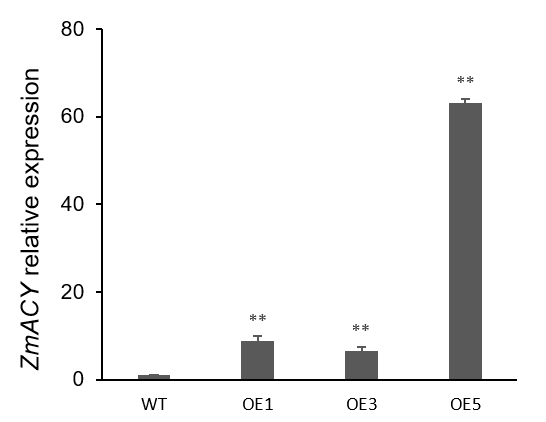

Supplement: Supplementary Figure 1 — Expression patterns of ZmACY in overexpressed lines. Wild-type and transgenic N. benthamiana were planted in a growth chamber at 23–26°C for 7 days. Three biological replicates were used. **P < 0.01. [file Image_1.tif]
