# Supplementary material for: ZmACY-1 Antagonistically Regulates Growth and Stress Responses in Nicotiana benthamiana
Source: Front Plant Sci. 2021 Jul 23;12:593001. doi: 10.3389/fpls.2021.593001 (PMC8343404; doi:10.3389/fpls.2021.593001)
Supplement: Supplementary Table 1 — Primers used in this study. [file Table_1.docx]

Supplementary table 1 Primers used in this study

| Primer | Sequence information |
| --- | --- |
| *ZmACY-1*-F | ATGCCGCCGCCGCCTCTCCGCTGT |
| *ZmACY-1*-R | TCAGCCTTGGAACGAGCTTAGTGC |
| qRT-*ZmACY-1*-F | AAGACATCGAGCAGATCAAGC |
| qRT-*ZmACY-1*-R | TCGCTGTGGATACGGGAC |
| qRT-Actin-F | GTCCATGAGGCCACGTACAA |
| qRT-Actin-R | CCGGACCAGTTTCGTCATA |
| XbaI-*ZmACY-1*-F | GCTCTAGAATGCCGCCGCCGCCTCTCCGCTGT |
| BamHI-*ZmACY-1*-R | CGGGATCCTCAGCCTTGGAACGAGCTTAGTGC |
| NbEF1a-F | CCTCAAGAAGGTTGGATACAAC |
| NbEF1a-R | TCTTGGGCTCATTAATCTGGTC |
| NbEXPA1-F | TTGTTTCTCTGCTTCTGGATGG |
| NbEXPA1-R | CTTAATGCAGCAGTGTTTGTACCA |
| NbEIN2-F | GGCATAATAGATCTGGCATTTTCC |
| NbEIN2 -R | TATCTAAGAGCATCGGTGCAGTTG |
| NbGS-F | TTGACCCCAAGCCTATTCCG |
| NbGS-R | TCCTCAAAGTATCCCTGTAGTTTGT |
| NbAS-F | GTCCACGGCATAAAAACGCC |
| NbAS-R | ACACAAAAGATGATCGTGGGA |
